# Software platform for flexible automated reconstruction of CMR data in a clinically feasible workflow

**DOI:** 10.1186/1532-429X-16-S1-W9

**Published:** 2014-01-16

**Authors:** Tamer A Basha, Sébastien Roujol, Kraig V Kissinger, Beth Goddu, Reza Nezafat

**Affiliations:** 1 Beth Israel Deaconess Medical Center, Boston, Massachusetts, USA

## Background

Integration of novel image reconstruction and processing methods into clinical workflow has been hampered by difficulties in implementing these techniques into the vendors' softwares. While all vendors allow modification of imaging sequences, implementation of novel reconstruction techniques is not available in any of them. Currently, the workflow includes manually exporting the data, performing the custom reconstruction using stand-alone Matlab or C++ implementation, and then visualizing the results on a different workstation. This process usually requires an expert user and is not feasible in common clinical workflow. In this work, we developed a simple software tool that enables researchers to rapidly integrate post-processing and reconstruction methods developed in any programming language on any type of workstation via internet connection and directly visualize the data on the scanner console and store the data via clinical PACS system.

## Methods

**Figure 1:**
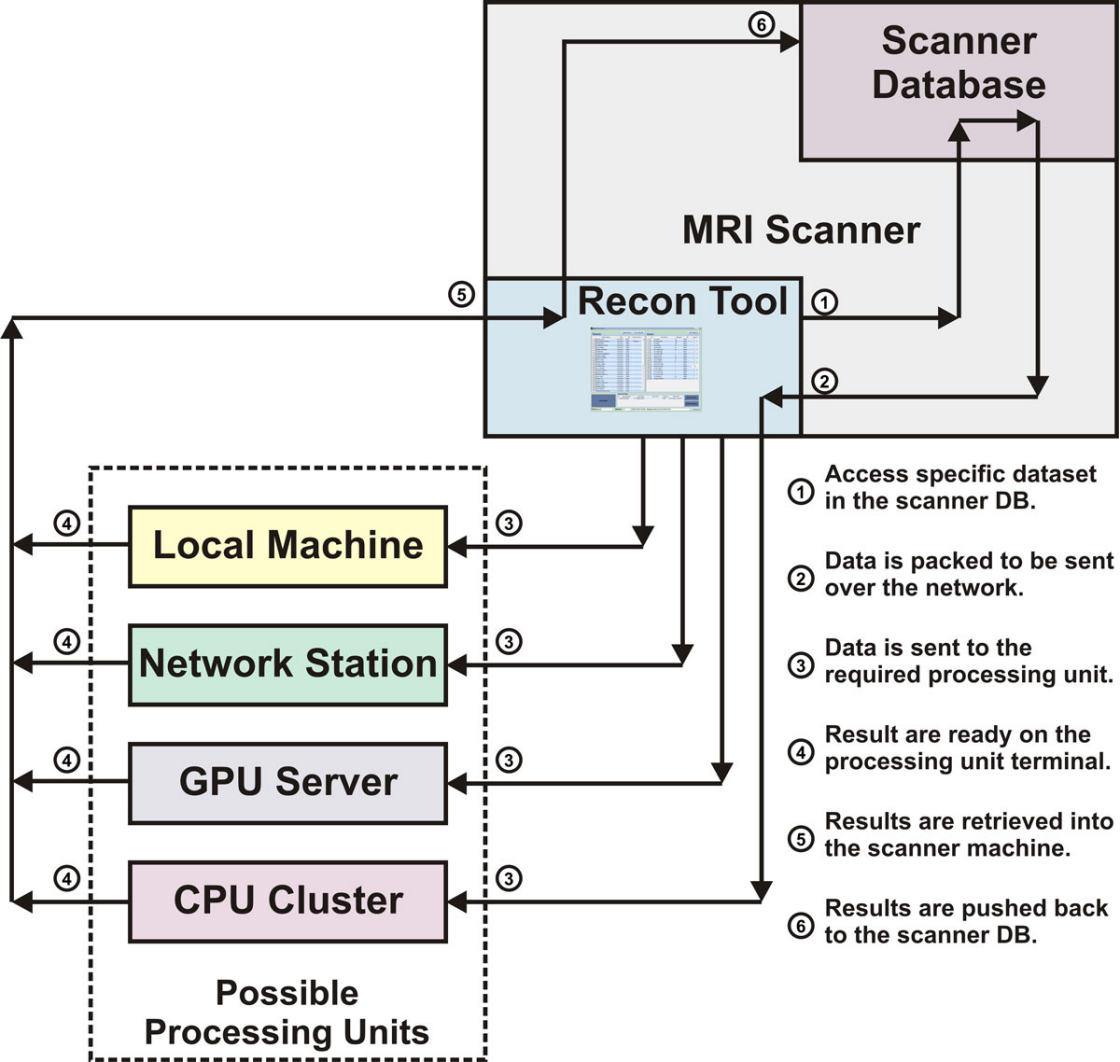
**Schematic diagram of the connections between the software program, scanner database and the processing units**. The circled numbers show the workflow steps for a processing algorithm to be applied on a dataset that resides on the scanner database.

Figure 1 shows a schematic diagram of the connections between the program, scanner and the remote processing units. The software program was developed in Matlab and is currently running on a Philips Achieva 1.5T scanner. After a scan is completed, the operator can invoke the program to initiate a specific reconstruction or processing method. The operator can choose the processing to occur on the scanner machine or on a different remote station on the network, or even on a CPU cluster or GPU server on the same network. During the processing, the program would connect with the remote processing workstation and update the progress of the operation. After finishing, the results are automatically retrieved from the remote processing unit and directly sent to the scanner database where they can be viewed and stored similar to images reconstructed by the vendor reconstruction system.

## Results

**Figure 2:**
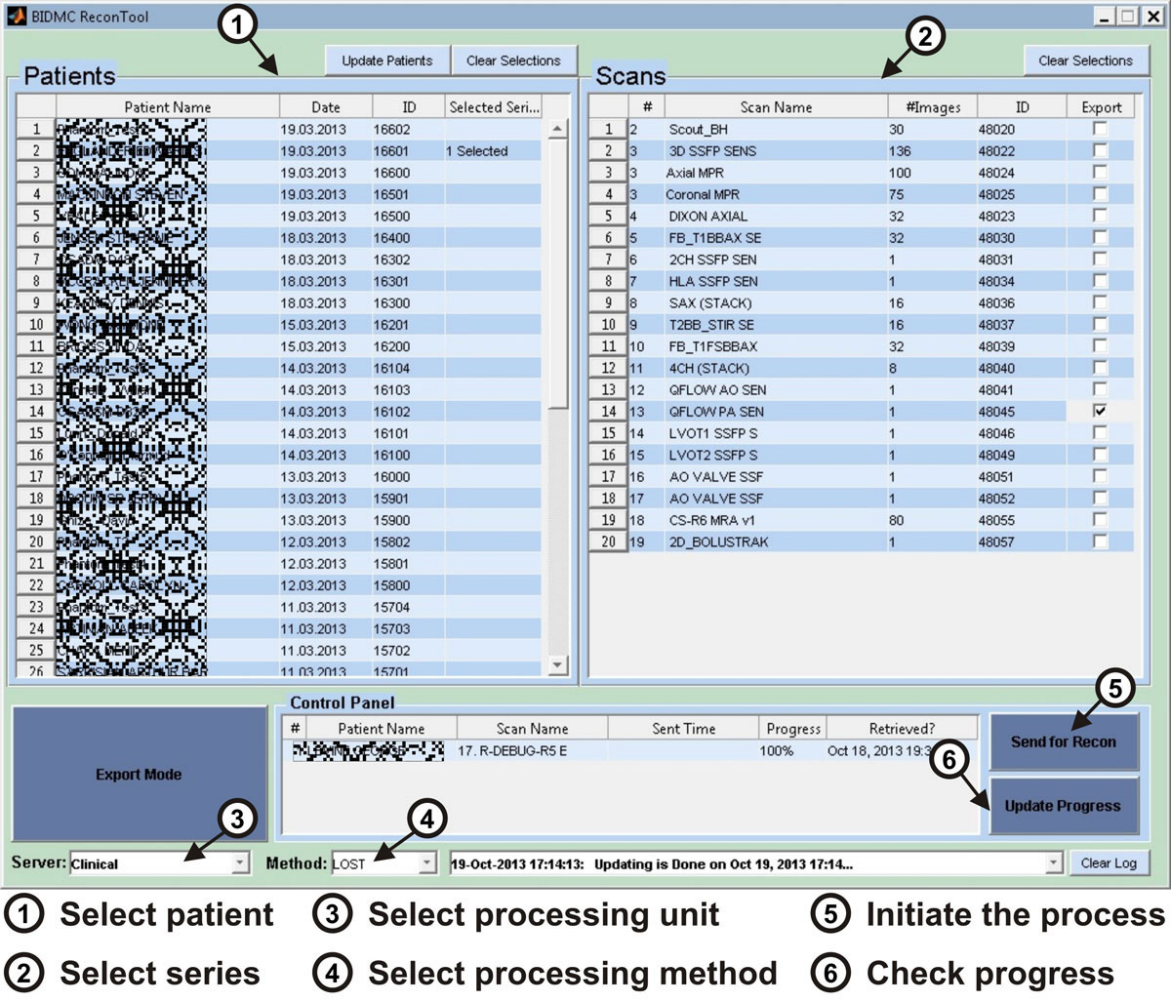
**The graphical user interface for the proposed software**. The circled numbers show the sequence of mouse clicks the operator has to do, in order to initiate a reconstruction/processing algorithm.

Figure 2 shows the user interface of the program that was intentionally designed with simple elements that require minimal operator interaction (Five mouse clicks to initiate and complete a full reconstruction method). This program is currently used by the MR technologists in our medical center to run advanced reconstruction techniques in their everyday practice. Such flexible software allows rapid integration of novel image reconstruction or processing methods to the clinical scans.

## Conclusions

We proposed and developed a software tool that allows researchers to test and validate different reconstruction/processing techniques in a smooth workflow combined with the MR machine.

## Funding

NIH: R01EB008743-01A2.

